# The Bridge Helix of RNA Polymerase Acts as a Central Nanomechanical Switchboard for Coordinating Catalysis and Substrate Movement

**DOI:** 10.1155/2011/608385

**Published:** 2012-01-22

**Authors:** Robert O. J. Weinzierl

**Affiliations:** Department of Life Sciences, Imperial College London, Sir Alexander Fleming Building, Exhibition Road, London SW7 2AZ, UK

## Abstract

The availability of *in vitro* assembly systems to produce recombinant archaeal RNA polymerases (RNAPs) offers one of the most powerful experimental tools for investigating the still relatively poorly understood molecular mechanisms underlying RNAP function. Over the last few years, we pioneered new robot-based high-throughput mutagenesis approaches to study structure/function relationships within various domains surrounding the catalytic center. The Bridge Helix domain, which appears in numerous X-ray structures as a 35-amino-acid-long alpha helix, coordinates the concerted movement of several other domains during catalysis through kinking of two discrete molecular hinges. Mutations affecting these kinking mechanisms have a direct effect on the specific catalytic activity of RNAP and can in some instances more than double it. Molecular dynamics simulations have established themselves as exceptionally useful for providing additional insights and detailed models to explain the underlying structural motions.

## 1. Introduction

RNA polymerases (RNAPs) are key enzymes of the cellular gene expression machineries of all organisms. Despite substantial progress during the last decade in elucidating high-resolution structures of RNAPs and the recent award of a Nobel Prize (Roger Kornberg, Chemistry 2006), there are still many unanswered questions regarding the mechanistic basis of transcription. This is mostly a consequence of the intrinsic complexity of the processes, but also due to a shortage of appropriate experimental data. Current models are predominantly shaped by the interpretation of X-ray crystal structures [1], but such approaches provide only a limited perspective. Crystallization trials require stable, catalytically inactive complexes as starting material, and many short-lived transitory conformations are unlikely to be preserved in crystal structures [2].

 During the last decade, we have pioneered alternative experimental strategies based on a hyperthermophilic archaeal system—the euryarchaeon *Methanocaldococcus jannaschii—*to devise an experimental system capable of generating functional insights in a systematic and high-throughput manner. We succeeded in creating an *in vitro* transcription system capable of promoter-specific transcription that consists entirely, including the RNAP, of recombinant proteins [3, 4]. Much of this work was guided by the key concept that the archaeal basal transcriptional machinery [5] closely mirrors the core components of the eukaryotic RNA polymerase II (RNAPII) system [6, 7], which is responsible for the highly regulated expression of all protein-encoding genes in eukaryotes. Archaeal RNAP subunits display extensive sequence homology to the eukaryotic subunits, and high-resolution structures of archaeal RNAPs are directly comparable to eukaryotic RNAPII [8, 9]. In addition, archaeal RNAPs use an identical set of basal factors to eukaryotic RNAPs to assist them with the sequence-specific initiation from promoters (TATA-binding protein and TFIIB; [10–16]). The archaeal basal transcriptional machinery thus encapsulates both structurally and functionally the essential core of the eukaryotic RNAPII transcriptional apparatus.

**Figure 1:**
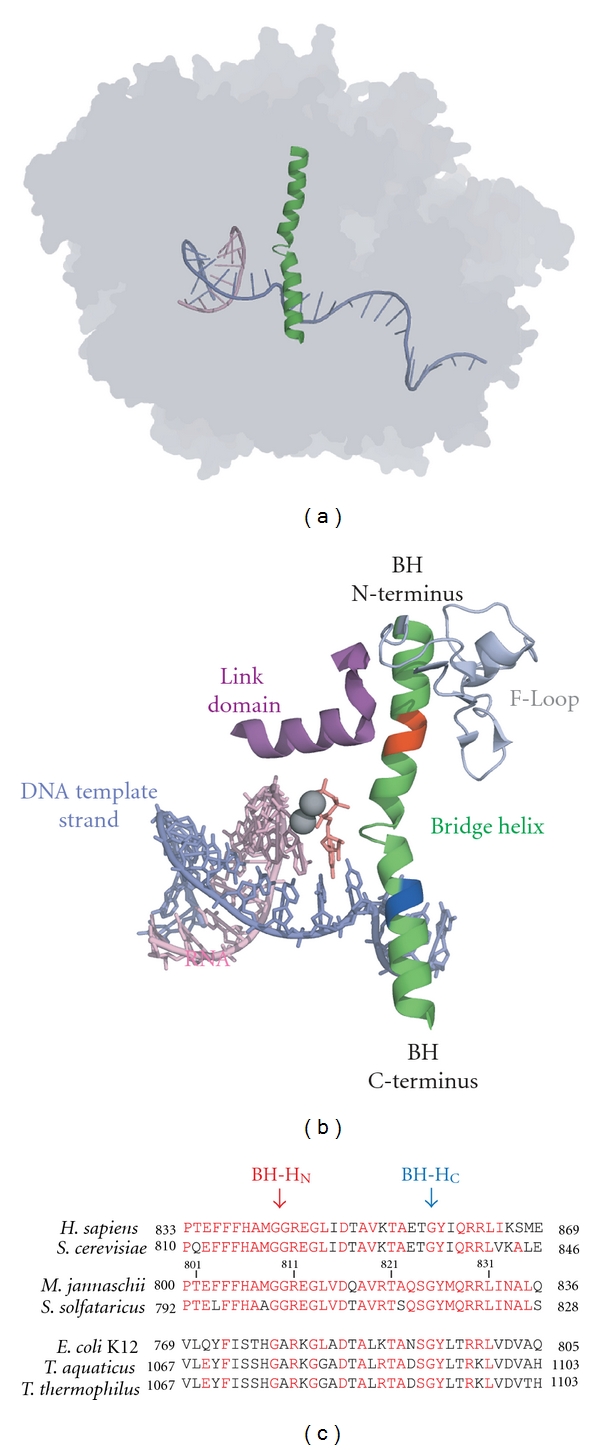
Structural aspects of the Bridge Helix. (a) Overall position of the Bridge Helix within RNAP. The Bridge Helix is shown as a green cartoon structure. Also shown are the DNA template strand (light blue) and the nascent transcript (pink). The remainder of the enzyme is shown as a transparent outline. Based on PDB# 2E2H [17] and visualized with PyMol [18]. (b) Detailed view of the Bridge Helix. The Bridge Helix is shown as a green cartoon structure, and the positions of the two molecular hinges are highlighted in red (BH-H_N_) and blue (BH-H_C_). Adjacent domains are shown in purple (Link Domain) or grey (F-Loop). The nucleic acid substrates are shown as stick models (DNA template strand, light blue; nascent transcript, pink; rNTP in insertion site, dark pink). The two grey spheres represent the magnesium ions (Metal-A and Metal-B) which represent the catalytic site. Based on PDB# 2E2H [17] and visualized with PyMol [18]. (c) Alignment of Bridge Helix sequences from eukaryotes (*Homo sapiens*; *Saccharomyces cerevisiae*), archaea (*Methanocaldococcus jannaschii* (euryarchaeota); *Sulfolobus solfataricus* (crenarchaeota)), and bacteria (*Escherichia coli*, *Thermus aquaticus*, and *Thermus thermophilus*). Residues that are identical to the reference organism used in the author's laboratory (*M. jannaschii*) are shown in red. The numbers flanking the sequences show the position of the Bridge Helix within the intact open reading frame of the subunits. The approximate locations of the two molecular hinge regions, BH-H_N_ and BH-H_C_, are indicated by arrows.

 Here, I will discuss in particular the importance of high-throughput approaches focused on the archaeal basal transcriptional machinery. The nature of molecular biological research has undergone a noticeable transition over the last decade. The rapid evolution of powerful experimental methodologies has shifted the traditional emphasis on individual genes and proteins to more wide-ranging aims, such as the large-scale gathering of comprehensive data sets [19]. Recognizing the opportunity to apply this systems-based philosophy to carry out an exhaustive mutagenesis screen of archaeal RNAPs, we recently automated the entire process of assembling recombinant archaeal RNAPs in large numbers on a robotic platform [20]. We have demonstrated the feasibility of high-throughput structure/function studies in a research program focused on the “Bridge Helix,” a 35-amino-acid-long *α*-helix that is the most prominent and highly conserved structure in the active site of all cellular RNAPs (Figure 1). The results show that the Bridge Helix domain is a conformationally versatile structural element that influences the functional properties of the catalytic center through a dynamic series of protein-protein and protein-nucleic acid interactions [2, 21–27]. The Bridge Helices found in archaeal RNAPs are very similar in sequence and structure to RNAPs from the two other evolutionary domains (Figure 1(c); [28, 29]), suggesting that many of the insights derived from archaeal model systems will be universally applicable across the RNAPs from the entire evolutionary range.

## 2. Functional Role of the Bridge Helix

The Bridge Helix is a central component of the catalytic site of all cellular RNAPs and intimately involved in all known functions of these enzymes (Figure 1(a)). The most basic function of RNAP is the DNA template-directed synthesis of transcripts which involves the successive extension of a nascent transcript by addition of nucleotide substrates. This process is thus frequently referred to as the “nucleotide addition cycle” (NAC). In the simplest form, the NAC depends on the precise coordination of a catalytic event (phosphodiester bond formation between the *α*-phosphate of an incoming rNTP and the 3′OH end of the nascent transcript) with the subsequent single-step translocation of the DNA-RNA hybrid away from the nucleotide insertion site to create space for the next nucleotide addition event. This process (catalysis-translocation) occurs cyclically for each nucleotide added to the transcript [1, 30–34]. Other events occurring further away from the catalytic site (e.g., separation of double-stranded DNA into template and nontemplate strands, separation of the transcript from the DNA, and reannealing of the DNA strands [35]) are similarly based on a series of temporary, but precise and energetically delicately balanced interactions between specific macromolecular surfaces. The NAC thus critically depends on coupling the completion of a catalytic reaction (phosphodiester bond formation) with precise nanomechanical movements of bulky nucleic acid substrates through the active site. Molecular machines—such as RNAPs—require a set of hinges and flexible loops that move domains to different positions at different stages of the reaction cycle, as well as sensor units that communicate completion of individual steps so that the enzyme can sequentially progress to the next step. As outlined below, the Bridge Helix appears to display a combination of many of the functional properties required to act as “nanomechanical switchboard” by combining physical translocation processes with substrate sensing functions.

## 3. Evidence for Bridge Helix Kinking

The view that the Bridge Helix contains nanomechanical hinges is based on multiple lines of evidence, including results obtained from X-ray crystallography, exhaustive site-directed mutagenesis, evolutionary conservation patterns and molecular dynamics analyses [21–27, 36–40]. Two sites in particular, which are referred to as Bridge Helix N-terminal Hinge (BH-H_N_) and C-terminal Hinge (BH-H_C_) [23], stand out as the most significant sites that are likely to undergo substantial conformational changes during the NAC. In the RNAP from the euryarchaeon *Methanocaldococcus jannaschii*, the helix-destabilizing imino acid proline can replace positions *mj*A' M808 and S824 without loss of catalytic activity and thus pinpoint the precise locations of BH-H_N_ and BH-H_C_ [21–24]. The naturally occurring primary amino acid sequences of both hinges are either highly conserved (BH-H_C_) or even essentially invariant (BH-H_N_) in all sequenced archaeal and eukaryotic polymerases. This confirms the functional importance of these hinges and suggests that the underlying primary amino acid sequences determine their key functional properties. Molecular dynamics simulations [41] have indeed revealed detailed insights that allow the formulation of plausible atomistic models for the hinge mechanisms: both BH-H_N_ and BH-H_C_ rely critically on one or more glycine residues that serve to destabilize the *α*-helical conformation in a geometrically highly localized manner [23, 27]. In BH-H_C_, the kink initiated at a single, evolutionary invariant glycine residue (*mj*A' G825) is subsequently most likely stabilized by cation-*π* interactions involving other nearby invariant residues (*mj*A'Y826 and R829/R830 [27]). In some species, there is evidence for a further electrostatic interaction providing additional stabilization of the kinked hinge conformation [39], but this is not a universally conserved feature [27]. Interestingly, the recently discovered RNAP IV and V enzymes [42] contain a naturally occurring proline residue in BH-H_C_ which is predicted to increase BH-H_C_ kinking (the physiological role of this unusual substitution is not yet understood).

 The molecular architecture of BH-H_N_ appears to make this hinge even more prone to kinking that BH-H_C_. This conclusion is based on the high sensitivity of a key residue (*mj*A' M808) to mutagenesis under *in vitro* conditions [23] but can also be deduced from the presence of three invariant glycine residues in close proximity to each other (*mj*A' G818, G819, and G822; Figure 1(c)), which causes a substantial regional weakening of the *α*-helical structure. Molecular dynamics simulations suggest that, similar to BH-H_C_, kinking of BH-H_N_ is initiated by unwinding of the *α*-helix in the glycine-containing segment. An energetically stabilized kink is then formed through van der Waal and hydrophobic interactions between the flanking side chains, most likely involving residues such as *mj*A' M808 and R820/E821 [23]. Interestingly, while the amino acid residues required for BH-H_C_ kinking are universally conserved in all organisms (bacteria, archaea, and eukaryotes), there appears to be a clearly discernible divergence in the structural features of BH-H_N_ between bacteria on the one hand and archaea/eukaryotes on the other. Keeping in mind what we know about the structure and function of archaeal/eukaryotic BH-H_N_, it appears that the bacterial BH-H_N_ regions are either less prone to kinking or do not kink in such a distinct manner. Molecular dynamics simulations of a bacterial RNAP suggest that bacterial Bridge Helices may kink more centrally [25] and possibly to a less significant extent. There is, however, also contrasting evidence compatible with the view that the position of bacterial BH-H_N_ may be directly comparable to the archaeal/eukaryotic species: a few bacterial species/isolates contain naturally occurring proline residues in the position orthologous to *mj*A' M808, that is, precisely the same place that tolerates a proline substitution in archaea [23]. It is therefore currently not entirely clear whether structural differences in bacterial BH-H_N_ motifs reflect a subtle difference in their mode of action. As described below, it seems very plausible that BH-H_N_ kinking is a key step in the NAC, so the precise location and function of bacterial BH-H_N_ sequences is an important question that needs to be experimentally addressed. 

## 4. Functional Implications of Bridge Helix Kinking during the Nucleotide Addition Cycle

The presence of two well-defined hinges in the Bridge Helix raises the question whether conformational changes in these hinges are likely to occur in the course of the NAC and, if yes, at what stage kinking may occur and what the functional consequences of such events might be. The only currently available crystal structures containing a kinked Bridge Helix (in BH-H_C_) have been crystallized in the complete absence of any substrates [36, 39] or complexed with an inhibitor capable of inducing an alternative structural state [40]. In contrast, inspection of X-ray structures of substrate-containing RNAPs gives the distinct impression that the Bridge Helix is firmly held in place by the nucleic acid substrates and nearby protein domains, thus reducing significantly any room available for substantial conformational changes (Figure 1(b)). Especially the BH-H_N_ region is surrounded by a variety of domains, such as the F-Loop, **β**-D, and Link domains [23] which appear to prevent any hinge movements. This impression of “not enough space to move” was certainly a major reason accounting for the rather belated discovery of BH-H_N_ because the Bridge Helix N-terminus appears consistently in strictly *α*-helical conformation in all bacterial, archaeal, and eukaryotic RNAP crystals characterized so far (e.g., [28, 29, 33, 35–40]).

 The comprehensive mutagenesis studies carried out on an archaeal Bridge Helix suggest, however, very strongly that the Bridge Helix hinges do not only exist but have a major effect on the catalytic rate of the RNAP. The increased specific activity (superactivity) that can be measured when particular residues are replaced by proline (*mj*A' M808 and S824; [21, 23]) suggests that in wildtype RNAPs the hinge movement may be a rate-limiting step that can be overcome by increasing the flexibilities of BH-H_N_ and BH-H_C_ [22]. These studies also suggest that increasing the flexibility of BH-H_N_ has an even more substantial effect than with BH-H_C_: a proline substitution of *mj*A' M808 (BH-H_N_) more than doubles the specific activity (~240% wildtype) as compared to a proline substitution of *mj*A' S824 (BH-H_C_) which increases the activity to a lesser extent (~170% of wildtype activity). Also, other mutations that stabilize the hinge in a kinked conformation increase the specific activity. The best example for this phenomenon is found in *mj*A' Q823 [21]. The *M. jannaschii* BH-H_C_ region is not naturally able to form the electrostatic bond that has been observed in kinked BH-H_C_ structures of other species (e.g., *T. aquaticus*; [39]) due the uncharged nature of *mj*A' Q823 [27]. A substitution of Q823 by either aspartic acid or, preferably, glutamic acid (*mj*A' Q823-D and Q823-E, resp.) results in the distinct levels of superactivity that are characteristic of a kinked conformation of BH-H_C_. Further evidence for the existence of these electrostatic interactions has been obtained by switching the positions of the charged residues [21]. 

 Taken together, either increases in the rate of hinge kinking (proline substitutions in BH-H_N_ and BH-H_C_) or increases in half-life of the kinked state (stabilization of electrostatic interactions in BH-H_C_) correlate strongly with a substantial increase in the rate of the NAC. It is thus reasonable to assume that Bridge Helix kinking is a naturally occurring process that plays an essential part during each cycle of the NAC. We need to address the nature of the conformational changes that occur within the catalytic site at various stages of the NAC and see how they could be affected by Bridge Helix kinking.

**Figure 2:**
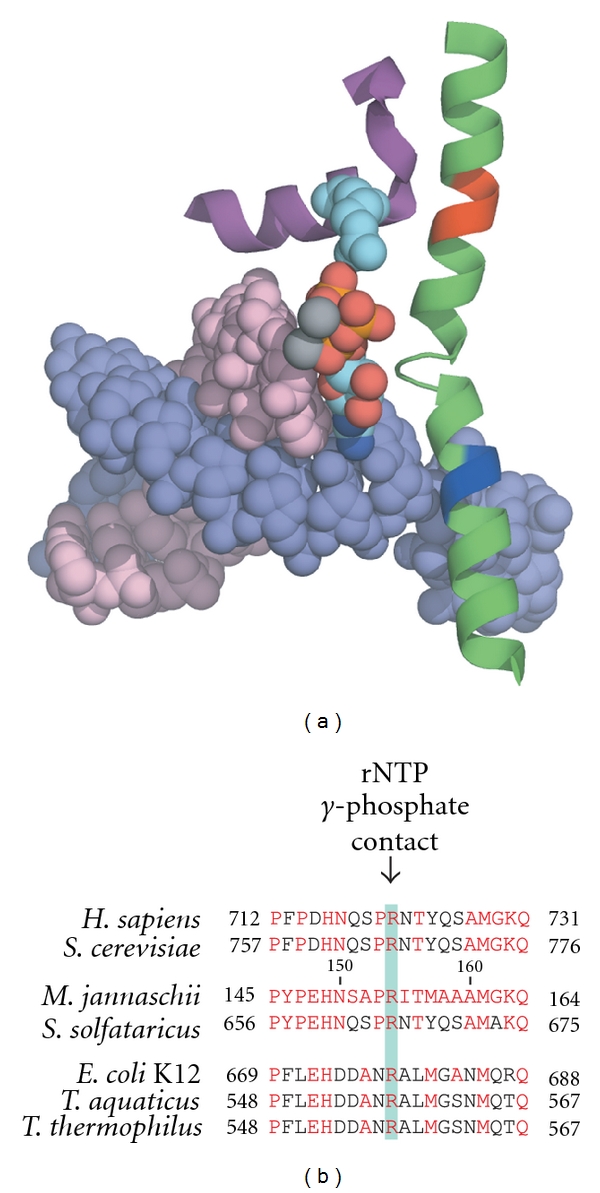
The Link domain. (a) Arrangement of the Bridge Helix and Link domain relative to the nucleotide insertion site. The Link domain (purple) and Bridge Helix domain (green) are shown as cartoon structures using the same coloring scheme as in Figure 1(b). The nucleic acid substrates are depicted in space-filling mode. Note the contacts made by the rNTP *γ*-phosphate (phosphate atoms in orange and oxygen atoms in red) with *sc*RPB2 R766 (shown in space-filling mode, light blue). (b) Alignment of Link domain sequences from the same species shown in Figure 1(c). The invariant arginine (R) is highlighted with a light green bar.

**Figure 3:**
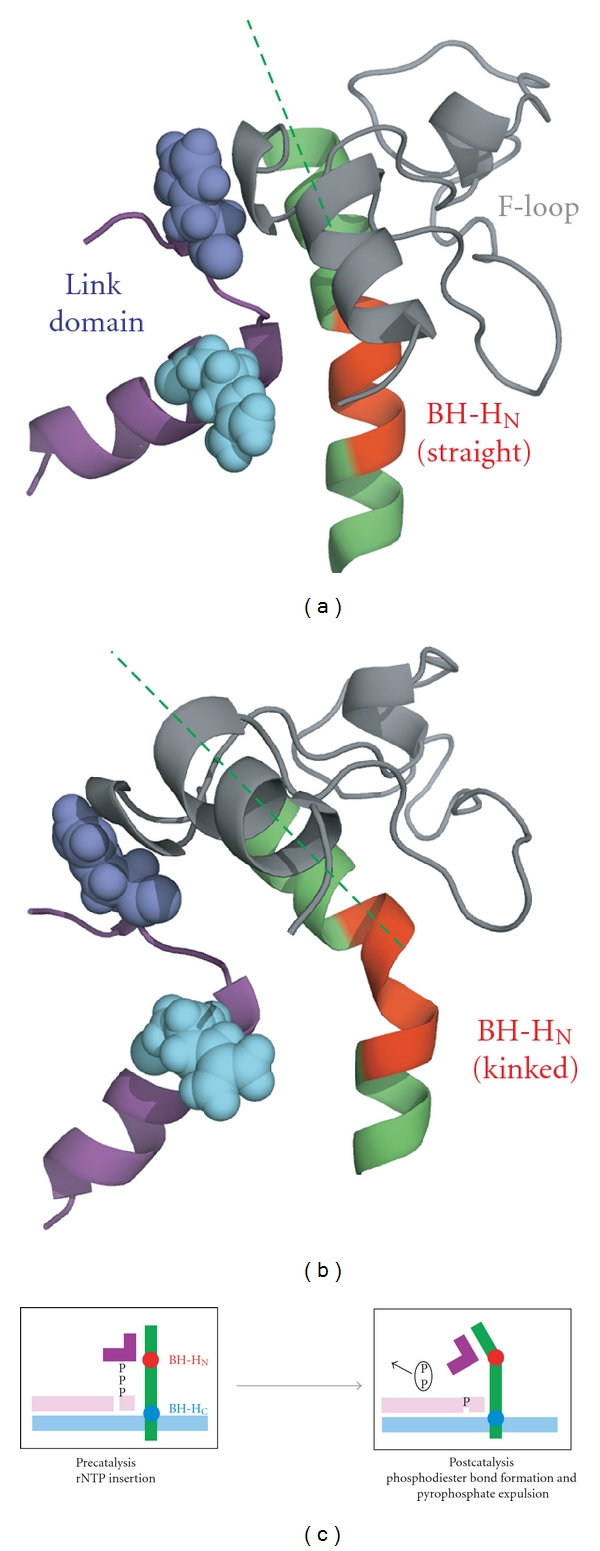
Structural connectivity between the F-Loop, Bridge Helix, and Link domain. (a) Structure of the complex at the begin of the simulation. The coloring scheme is as described in Figure 2(a). An additional residue, *sc*RPB2 H761, making close contact to the F-Loop is shown in space-filling mode in dark blue. The Bridge Helix N-terminus is fully *α*-helical and essentially straight. (b) During the molecular dynamics simulation, a kink in BH-HN (red section of the Bridge Helix) occurs and tilts the F-Loop and associated Link domain towards the left. (c) Interpretation of the effects of the structural changes observed in (a) and (b) on the nucleotide insertion site. The removal of the pyrophosphate group after successful incorporation of the nucleotide into the nascent transcript removes a structurally stabilizing contact and may thus influence the spatial position and conformation of the Link domain.

In the absence of additional crystal structures displaying kinked Bridge Helices, we have to rely primarily on further site-directed mutagenesis studies of the surrounding domains to reveal further clues of the conformational changes that may occur at particular catalytic stages. A particularly intriguing small domain, the Link domain (see Figure 1(b) for the location of this structure), could play an important role in establishing a functional connection between the catalytic site and the N-terminal part of the Bridge Helix [23]. The Link domain is L-shaped and apparently provides an indirect conformational link between the rNTP in the insertion site and the Bridge Helix N-terminus (Figure 2(a)). Electrostatic contacts between the *γ*-phosphate of the rNTP and an evolutionarily invariant arginine residue (Figure 2(b); *sc*RPB2 R766 in yeast RNAPII corresponding to *mj*B' R154 in *M. jannaschii* RNAP) are likely to stabilize the binding of the rNTP at the insertion site. This interaction could, however, have an equally important function as a molecular sensor to communicate the occupation of the insertion site by an rNTP to the Bridge Helix. In yet unpublished work, we have studied the structural connectivity of the Link domain to the Bridge Helix using molecular dynamics simulations. The results show that contacts between the Bridge Helix and Link domain depend critically on the presence of the F-Loop [43], which is a cap-like extension of the N-terminus of the Bridge Helix (Figure 1(b)). The presence of the F-Loop and Link domain does not interfere with BH-H_N_ kinking properties and demonstrates that the entire Bridge Helix N-terminus/F-Loop/Link domain complex appears to move as a single rigid body (Figures 3(a) and 3(b); ROJW, manuscript in preparation). Although this concept requires further experimental verification, it can be imagined that such a mechanism could serve as a conformational sensor that induces BH-H_N_ kinking after successful phosphodiester bond formation and pyrophosphate release (Figure 3(c)).

## 5. Outlook

We are currently at a stage where we begin to discern the major outline of the mechanistic basis of the NAC [1] but still lack many of the most relevant details for describing the sequence of conformational changes that are either known to occur within the catalytic site or can be inferred from a variety of other observations. Although it would be fascinating to obtain high-resolution X-ray models of RNAPs containing kinked Bridge Helices, the relative lack of success thus far shows that such structures cannot be crystallized routinely (even the two examples of bacterial RNAPs with BH-H_C_ kinks [36, 39] turned out to be exceptional and similar crystals have not been found in more recent crystallization trials [D. Vassylyev; pers. comm.]). It therefore seems likely that further investigations of the structural changes of the Bridge Helix and the way such alternative conformations influence the processes in the active site at different stages of the NAC will to a large extent be driven by further high-throughput mutagenesis studies and molecular dynamics simulations [23, 25, 27, 41]. The already existing archaeal model systems are thus ideally placed to continue to make substantial contributions towards a detailed understanding of this fundamental biological process in the foreseeable future.
